# Mortality and associated risk factors in perioperative acute kidney injury treated with continuous renal replacement therapy

**DOI:** 10.1186/s13741-021-00227-y

**Published:** 2021-12-14

**Authors:** Panu Uusalo, Tapio Hellman, Mikko J. Järvisalo

**Affiliations:** 1 Department of Anaesthesiology and Intensive Care, University of Turku, P.O. Box 51, Kiinamyllynkatu 4-8, FI-20521 Turku, Finland; 2 Perioperative Services, Intensive Care and Pain Medicine, Turku University Hospital, Turku, Finland; 3 Kidney Center, Turku University Hospital, Turku, Finland

## Abstract

**Background:**

Perioperative acute kidney injury (AKI) is associated with multiple postoperative complications leading to prolonged hospital stay and higher costs. AKI requiring continuous renal replacement therapy (CRRT) after surgery has an incidence of 2–6% and mortality approximates 40–60%. Previous studies examining mortality in perioperative AKI patients managed with CRRT have concentrated on cardiac surgery patients and there are very limited data on broad surgical patient populations requiring CRRT. We examined long-term mortality and factors associated with poor outcome in a broad surgical population requiring CRRT for perioperative AKI during a 10-year period.

**Methods:**

Surgical patients admitted to the intensive care unit (ICU) of academic tertiary hospital requiring CRRT between years 2010–2019 were included. CRRT was performed using regional citrate-calcium-anticoagulation. Extracted data included patient demographics, comorbidities, and clinical parameters at ICU admission and at the initiation of CRRT. Creatinine and estimated glomerular filtration rate (eGFR) were measured at 1 year after ICU admission.

**Results:**

A total of 157 patients were included in the study. ICU mortality was 42.7%, 90-day mortality 58.0% and 1-year mortality 62.4%. Blood lactate at ICU admission and CRRT initiation were independently associated with mortality in the multivariate models. Patients with lactate > 4 mmol/l had higher mortality than patients with normal lactate (77% vs. 21%) (*p* < 0.001). Creatinine (*p* = 0.004) and eGFR (*p* < 0.001) remained significantly altered at 1 year of follow-up compared to baseline.

**Conclusions:**

Patients undergoing surgery and requiring perioperative CRRT in the ICU have a high risk of mortality. Mortality appears to be independently associated with lactate levels.

**Supplementary Information:**

The online version contains supplementary material available at 10.1186/s13741-021-00227-y.

## Introduction

Major surgery is among the most common risk factors for acute kidney injury (AKI) (Hobson et al., 2009). Furthermore, perioperative AKI is associated with increased risk of sepsis, anemia, coagulopathy, and mechanical ventilation (Bihorac et al., 2009). Particularly, patients with severe comorbidities such as coronary artery disease, diabetes, and patients with high American Society of Anesthesiologists physical status are at high risk for developing perioperative AKI (Biteker et al., 2014).

Continuous renal replacement therapy (CRRT) potently extracts lactate, corrects acid base imbalance and other metabolic complications of perioperative AKI (Bagshaw et al., 2017; Tandukar & Palevsky, 2018) as well as removes fluid overload, a known risk factor for mortality (Hall et al., 2020). Despite CRRT, the risk for mortality and chronic kidney disease remains high in perioperative AKI patients (Goren & Matot, 2015). Moreover, CRRT is expensive, carries a high staff and equipment requirement, and is labor intensive (Hoyt, 1997).

A vast majority of previous studies examining mortality in perioperative AKI patients managed with CRRT have concentrated on cardiac surgery patients and several studies have shown that blood lactate is associated with mortality after cardiac surgery (Maillet et al., 2003; Minton & Sidebotham, 2017). However, there are very limited data on factors associated with mortality in broad surgical patient populations requiring CRRT. Thus, further studies on individual risk assessment and prognosis of surgical intensive care unit (ICU) patients requiring CRRT are warranted.

We aimed to study ICU and long-term mortality and potential risk factor associations including blood lactate levels in a broad surgical population requiring CRRT for perioperative AKI during a 10-year period.

## Methods

### Data sources, collection, and study population

Patients undergoing surgery and admitted to a single ICU of an academic tertiary medical center from 1 January 2010 through 31 December 2019 and requiring CRRT were included in this retrospective cohort study. The individual patient data were collected from the hospital’s medical documents with the permission of the University Hospital Clinical Research Center scientific review board and the Hospital district of Southwest Finland. The patient identity numbers were removed before the statistical analyses. For this retrospective, register-based, non-interventional study the regulatory review board waived the need for informed consent in terms of collection and analysis and publication of data.

For the purpose of this study, blood pH, bicarbonate, lactate, base excess, electrolytes, and other laboratory variables, blood pressure, need for invasive mechanical ventilation, PaO2/FiO2-ratio, diuresis, and vasopressors were recorded at ICU admission and at CRRT initiation. Other data extracted from patients’ medical records included demographics, chronic medical conditions, fluid balance at CRRT initiation, CRRT dose and Acute Physiology and Chronic Health Evaluation (APACHE) II score, Simplified Acute Physiology Score (SAPS) II, and Sequential Organ Failure Assessment (SOFA) score. Creatinine and estimated glomerular filtration rate (eGFR) were assessed at baseline, within 1 year prior to ICU admission as available and 1 year after discharge from the ICU in surviving patients.

### CRRT modality

Continuous Veno-Venous Hemodialysis for all patients was performed using Fresenius Multifiltrate CRRT monitors and 1.80 m^2^ polysulfone hemofilters Ultraflux AV1000 or Ultraflux EMiC2 HCO membranes with CiCa dialysate to achieve regional citrate anticoagulation (Fresenius Medical Care, Bad Hamburg, Germany). Post-filter-ionized calcium levels were used for anticoagulation monitoring. Blood and dialysate flow rates were prescribed according to the weight of the patient and by the caring ICU physician to target a dialysis dose of > 25 ml/kg/h. The methodology for CRRT remained unaltered for the entire study period.

### Statistical analysis

Results are presented as mean ± standard deviation (SD) for the normally distributed variables and as median inter-quartile range (IQR) for skewed variables. Normality in continuous covariates was tested with Kolmogorov-Smirnov and Shapiro-Wilk tests. Student’s *t* test was used to compare continuous normally distributed covariates and chi-square test for categorical covariates in the study subgroups. For variables with skewed distributions, groupwise comparisons were done using a non-parametric Kruskal-Wallis test. Comparisons between creatinine and eGFR, respectively, at baseline and at 1 year were done using paired *t* tests.

The relationship between mortality and exposure variables of interest were examined using univariable and stepwise multivariable Cox proportional hazard models. Variables that were significantly associated with mortality in univariate Cox models were included as covariates in two respective stepwise multivariable Cox proportional hazards models. To avoid significant collinearity in the models in terms of laboratory and clinical data at ICU admission and CRRT initiation, multivariable analyses were performed by using two respective stepwise multivariable

### Cox models

One including significant baseline characteristics and laboratory variables at ICU admission and one with baseline characteristics and variables at CRRT initiation.

All statistical analyses were performed using statistical analysis system, SAS version 9.3 (SAS Institute Inc., Cary NC). *P* < 0.05 was considered statistically significant.

### Ethics

The study protocol was approved by the Hospital District of Southwest Finland (T146/2016).

This was a retrospective register-based study of patients from an anonymized dataset that only involved recording data from medical records. According to Finnish law and Ethics committee of South-West Finland Hospital District this study did not require consent from patients to participate. All data was anonymized, and this study does not contain any individual person’s data in any form (including individual details, images or videos). Therefore, consent for publication was waived.

## Results

### Patient characteristics

A total of 9655 patients underwent surgery and received postoperative ICU care between January 2010 and December 2019. Overall, 165 (1.7%) patients required CRRT. Eight patients on maintenance dialysis were excluded leaving 157 patients (44 women, 28.0%) with a mean age of 68.7 ± 11.5 years for the analyses. The most common comorbidities were hypertension (69%) and coronary artery disease (37%) (Table 1). Prior medications are shown in Supplement Table 1. Largest surgical groups were cardiac surgery (44.6%), vascular surgery (24.2%) and gastrointestinal surgery (19.1%). Of the patients undergoing cardiac surgery 17 (24%) underwent coronary artery bypass grafting (CABG), 12 (17%) single valve replacement, 12 (17%) single valve replacement with CABG, 12 (17%) aortic arch replacement, 8 (11%) acute aortic dissection correction, 6 (9%) double valve replacement, and 3 (4%) double valve replacement with CABG. Mean duration of cardiopulmonary bypass was 182±101 min. Almost all patients (98.7%) required vasopressor support at ICU admission and (94.3%) required mechanical ventilation during ICU stay. Median (IQR) duration of mechanical ventilation was 7.9 (2.9–13.1) and tracheostomy was required in 31 (19.8%) patients (Table 1). One hundred and twenty-four (79%) patients were operated on the day of ICU admission.

**Table 1:** Baseline patient characteristics and mortality of the 157 patients

Women (n/%)	44/28.0
Age (years)	68.7 ± 11.5
Diabetes (*n*/%)	36/23
Hypertension (*n*/%)	109/69
Pulmorary disease (*n*/%)	20/13
Coronary artery disease (*n*/%)	61/39
Peripheral arterial disease (*n*/%)	23/15
Liver cirrhosis (*n*/%)	1/1
Malignancy (*n*/%)	12/8
Peak SOFA	14.9 ± 2.9
SAPS-II	51.9 ± 13.3
APACHE-II	23.8 ± 6.5
ICU stay (days, survivors, *n* = 90)	17.3 ± 15.1
Elective (*n*/%)	44/28.0
Cardiac surgery (*n*/%)	70/44.6
Gastrointestinal surgery (*n*/%)	30/19.1
Vascular surgery (*n*/%)	38/24.2
Trauma surgery (*n*/%)	7/4.5
Other surgery^a^ (*n*/%)	12/14.9
Requiring mechanical ventilation (*n*/%)	155/98.7
Days on mechanical ventilation (days)	7.9 (2.6–13.1)
Vasopressor use (*n*/%)	155/98.7
Baseline creatinine, *n* = 137 (μmol/l)	88 (69–115)
Baseline eGFR, *n* = 137 (ml/min/1.73 m^2^)	78 ± 24
ICU mortality (*n*/%)	67/42.7
90 day mortality (*n*/%)	91/58.0
One year mortality (*n*/%)	98/62.8

*SOFA* Sequential Organ Failure Assessment Score, *SAPS-II* Simplified Acute Physiology Score, *APACHE-II* Acute Physiology And Chronic Health Evaluation Score II, *ICU* intensive care unit, *eGFR* = estimated glomerular filtration rate

Values are mean ± SD or median (IQR).

^a^Other surgery includes urology *n* = 2, orthopedic surgery *n* = 4, gynecological surgery *n* = 1, and plastic surgery *n* = 4 patients

Fifty-seven (36%) patients were observed with sepsis during ICU care. The source of infection was abdominal in 25 (43%), skin/soft tissue in 15 (26%), blood-borne in 8 (14%), respiratory in 7 (12%) and urinary in 2 (4%) patients. Most common antimicrobials used in patients with sepsis were meropenem (19%), piperacillin-tazobactam (11%), and fluconazole (9%). The specific antimicrobial regimens used in patients with sepsis are listed in Supplemental Table 1.

### Determinants of mortality

**Fig. 1:**
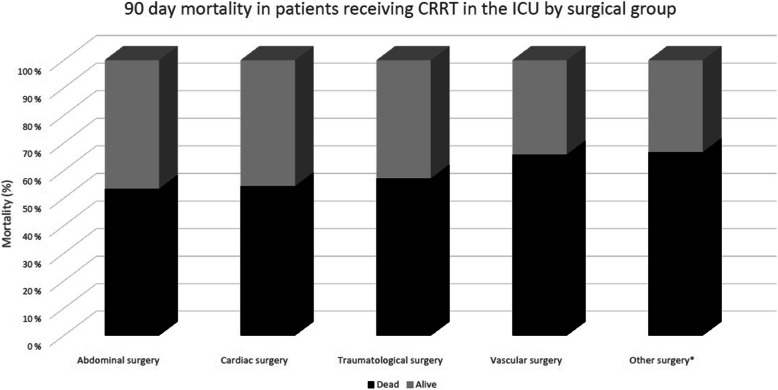
90-day mortality in patients receiving CRRT in the ICU by surgical group

Patients were followed up for a mean of 756 ± 1125 days. One hundred fourteen patients (73%) died during follow-up. ICU mortality was 42.7%, 90-day mortality 58.0% and 1-year mortality 62.4%. The 90-day mortality rate was similar across the different surgical groups (Fig. 1).

The ICU survivors had a similar amount of operation room visits and similar incidence of sepsis compared with patients who died during ICU care. There were no differences in pH, CRP, hemoglobin, leukocytes, thrombocytes, urea, bilirubin, sodium, potassium, chloride, ionized calcium, mean arterial pressure, or norepinephrine dose at the time of ICU admission. However, non-survivors had lower creatinine, base excess and bicarbonate at time of ICU admission, and higher age, blood lactate, international normalized ratio, peak SOFA, SAPS-II, APACHE-II, number of required vasopressors, and maximum norepinephrine dose (Table 2).

**Table 2:** Characteristics of ICU-survivors compared to non-survivors

Variable	Survivors (***n*** = 90)	Non-survivors (***n*** = 67)	***p*** value
Age (years)	66.7 ± 12.4	71.2 ± 9.7	0.01
Women *n*/%	19/21	25/37	0.025
Hypertension *n*/%	59/66	50/75	0.22
Diabetes *n*/%	24/27	12/18	0.20
Pulmonary disease *n*/%	11/12	9/13	0.82
Coronary artery disease *n*/%	29/32	32/48	0.048
Peripheral artery disease *n*/%	9/10	14/21	0.06
Malignancy *n*/%	10/11	2/3	0.06
Peak SOFA	14.4 ± 3.1	15.7 ± 2.6	0.008
SAPS-II	49.2 ± 13.7	55.7 ± 11.7	0.002
APACHE-II	22.1 ± 6.7	26.2 ± 5.3	< 0.0001
Elective operation (*n*/%)	22/24.4	22/32.8	0.25
Operation room visits	2 (1–3)	2 (1–2)	0.25
Sepsis (*n*/%)	31/34.4	26/38.8	0.57
Ventilator days	8.9 (4.9–13.9)	4.9 (1.5–9.8)	0.001
Hemoglobin (g/l)	98 (90–114)	100 (88–111)	0.60
Leukocytes (E^9^/l)	11.4 (8.3–16.45)	10.6 (8.1–17.3)	0.86
Thrombocytes (E^9^/l)	115 (89–170)	111 (83–163)	0.50
C-reactive protein (mg/l)	92 (55–190)	53 (24–214)	0.14
Creatinine (μmol/l)	191 (122–279)	151 (119–202)	0.08
Urea (mmol/l)	10.6 (7.5–18.6)	9.8 (6.7–17.1)	0.60
Alanine aminotransferase (IU/l)	33 (18–83)	25 (14–63)	0.41
Bilirubin (μmol/l)	15 (9–30)	18 (11–39)	0.41
International normalized ratio	1.4 (1.2–1.7)	1.5 (1.3–1.8)	0.16
pH (U)	7.32 (7.26–7.36)	7.31 (7.22–7.35)	0.06
BE (mmol/l)	− 5.4 (− 8.8 to − 3.2)	− 7.2 (− 11.0 to − 3.3)	0.06
Bicarbonate (mmol/l)	19.7 ± 4.0	18.4 ± 4.0	0.04
Lactate (mmol/l)	2.5 (1.6–4.2)	4.2 (2.3–7.4)	0.0002
Sodium (mmol/l)	136 ± 4	137 ± 3	0.11
Potassium (mmol/l)	4.4 (3.9–4.9)	4.3 (3.9–4.8)	0.39
Chloride (mmol/l)	109 ± 4.7	109 ± 4.3	0.83
Ionized calcium (mmol/l)	1.10 (1.04–1.14)	1.09 (1.02–1.16)	0.89
Mean arterial pressure (mmHg)	73 ± 15	70 ± 12	0.09
Norepinephrine dose (μg/kg/min)	0.10 (0.03–0.16)	0.12 (0.03–0.28)	0.09
Maximum norepinephrine dose (μg/kg/min)	0.20 (0.13–0.33)	0.33 (0.20–0.53)	< 0.0001
Number of vasopressors (*n*)	1 (1–2)	2 (1–3)	0.03

*SOFA* Sequential Organ Failure Assessment Score, *SAPS-II* Simplified Acute Physiology Score, *APACHE-II* Acute Physiology And Chronic Health Evaluation Score II, *RBC* red blood cell

Values are mean ± SD or median (IQR)

Laboratory values are given at ICU admission, unless stated otherwise

**Fig. 2:**
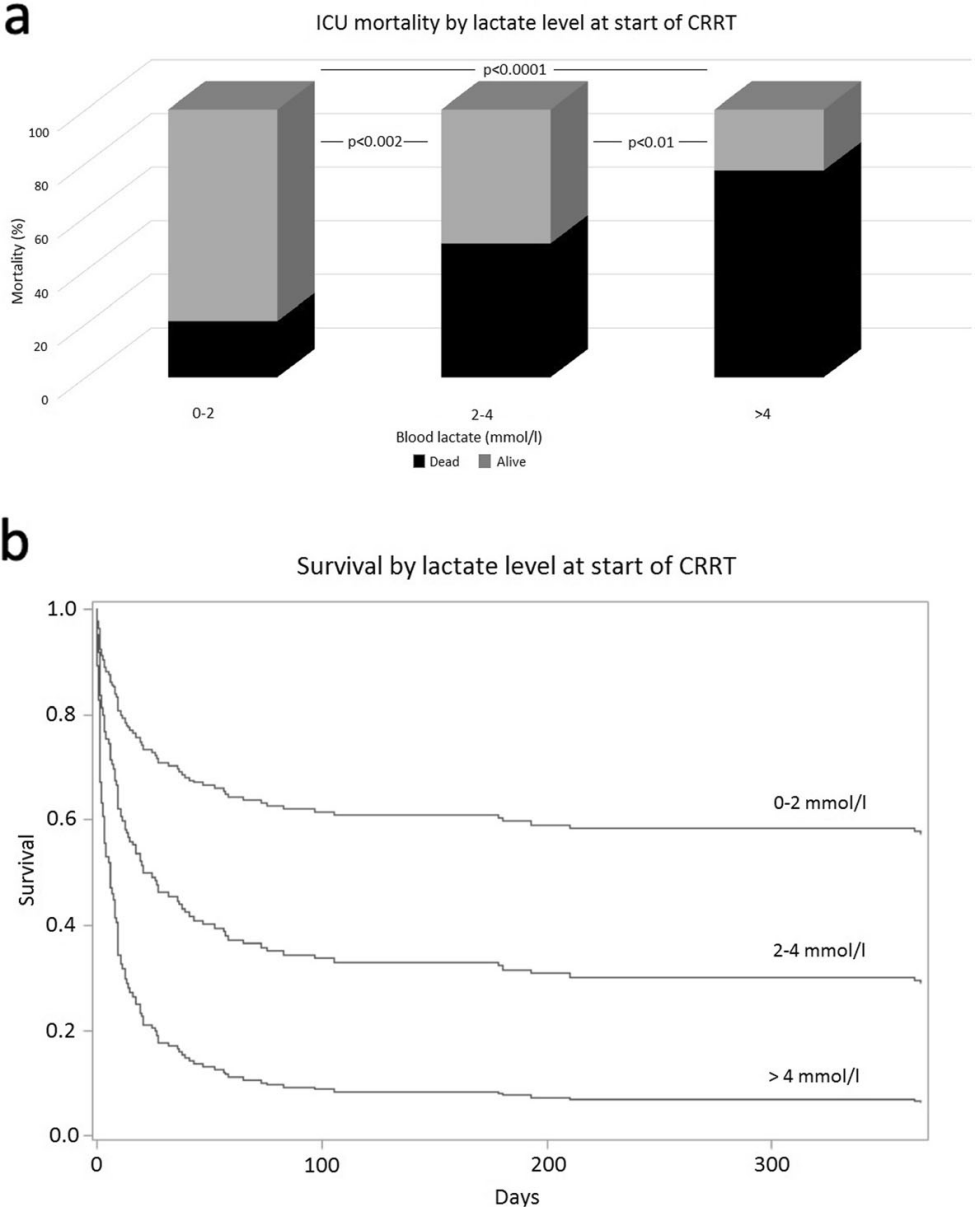
ICU mortality rates (**a**) and probability of survival (**b**) according to lactate level at the start of CRRT

There were no differences in the delay from ICU admission to CRRT initiation or dialysis dose between survivors and non-survivors. Mean arterial pressure, fluid balance and urea at the time of CRRT initiation were similar between the groups. However, survivors had higher pH, PaO2/FiO2-ratio, and creatinine at CRRT initiation compared to non-survivors, whereas non-survivors had higher potassium, lactate, and norepinephrine dosing requirement (Table 3). The ICU mortality rate was higher in the patients with mild lactatemia (2–4 mmol/l) and more severe lactatemia (> 4 mmol/l) compared with patients with normal lactate at the initiation of CRRT (50% vs 21% and 77% vs 21%, *p* ≤ 0.01 for both comparisons), respectively (Fig. 2).

**Table 3:** Parameters at the initiation of CRRT by survivor group

Variable	Survivors (***n*** = 90)	Non-survivors (***n*** = 67)	***p*** value
CRRT initiation after ICU admission (days)	1.23 (0.60–2.79)	1.05 (0.51–2.17)	0.44
Dialysis dose (ml/kg/h)	33.9 (29.3–36.0)	34.7 (32.7–37.5)	0.06
Creatinine (μmol/l)	267 (179–367)	213 (148–278)	0.005
Urea (mmol/l)	15.9 (9.8–22.7)	12.6 (8.7–20.3)	0.14
Potassium (mmol/l)	4.2 ± 0.6	4.4 ± 0.8	0.02
pH (U)	7.34 ± 0.08	7.24 ± 0.13	< 0.001
Bicarbonate (mmol/l)	20.7 (18.5–22.0)	16.6 (14.2–21.0)	< 0.001
Lactate (mmol/l)	1.44 (1.10–2.37)	4.50 (1.88–9.33)	< 0.001
Fluid balance (ml)	3115 (1075–5689)	3343 (1328–5981)	0.60
Diuresis (ml/kg/h)	0.20 (0.07–0.64)	0.10 (0.04–0.33)	0.03
Norepinephrine requirement (μg/kg/min)	0.095 (0.03–0.20)	0.22 (0.11–0.36)	< 0.001
Mean arterial pressure (mmHg)	70 (65–80)	68 (63–75)	0.07
PaO2/FiO2 (kPa)	27 (19–37)	22 (15–31)	0.01

*CRRT* continuous renal replacement therapy, *ICU* intensive care unit, *MAP* = mean arterial pressure, *PaO2/FiO2* = ratio of arterial oxygen partial pressure to fractional inspired oxygen

Values are mean ± SD or median (IQR)

Factors associated with mortality were assessed using univariate and stepwise multivariate Cox proportional hazards models. Variables with significant univariate associations with mortality and thereby included in the respective multivariate models are shown in Table 4.

**Table 4:** Univariable predictors of all-cause mortality

Variable	Unadjusted HR (95% CI)	*p*-value
Age (years)	1.027 (1.01-1.05)	0.002
Female	0.63 (0.43-0.94)	0.02
APACHE-II	1.06 (1.03-1.09)	<0.001
SAPS-II	1.03 (1.02-1.05)	<0.001
Hypertension	1.53 (1.01-2.34)	0.045
Coronary artery disease	1.50 (1.03-2.14)	0.03
Peripheral artery disease	1.97 (1.22-3.17)	0.005
Lactate at ICU admission (mmol/l)	1.080 (1.04-1.12)	<0.001
Lactate at start of CRRT (mmol/l)	1.19 (1.14-1.24)	<0.001
INR at ICU admission	1.45 (1.04-2.02)	0.03
Hemoglobin at start of CRRT	1.01 (1.00-1.02)	0.045
Creatinine at start of CRRT	0.996 (0.994-0.998)	<0.001
CRP at start of CRRT	0.997 (0.996-0.999)	0.007
pH at start of CRRT (per 0.1)	0.94 (0.92-0.96)	<0.001
BE at start of CRRT	0.89 (0.85-0.93)	<0.001
Bicarbonate at start of CRRT	0.86 (0.81-0.91)	<0.001
PaO2/FiO2-ratio at start of CRRT	0.98 (0.97-1.00)	0.02
Highest noradrenaline requirement (ug/kg/min)	1.19 (1.09-1.29)	<0.001
Noradrenaline requirement at ICU admission (ug/kg/min)	1.15 (1.03-1.29)	<0.001
Noradrenaline requirement at start of CRRT (ug/kg/min)	1.32 (1.20-1.47)	<0.001
Number of vasopressors	1.25 (1.02-1.54)	0.04

Vasopressor dose includes a combined dose of norepinephrine, epinephrine and vasopressin

APACHE-II = Acute Physiology and Chronic Health Evaluation Score II; SAPS-II = Simplified Acute Physiology Score; CRRT = Continuous Renal Replacement Therapy; ICU = Intensive Care Unit; BE = base excess; PaO2/FiO2 = ratio of arterial oxygen partial pressure to fractional inspired oxygen

Due to significant collinearity in the laboratory and clinical data at ICU admission and CRRT initiation, multivariable analyses were performed using two respective stepwise multivariable Cox models: one including significant baseline characteristics and laboratory variables and one with baseline characteristics and variables at CRRT initiation. Altogether, SAPS-II [per 1 point, hazard ratio 1.027 (95% CI 1.009–1.045), *p* = 0.003], age [per year, hazard ratio 1.020 (95% CI 1.001–1.040), *p* = 0.041], coronary artery disease [hazard ratio 1.548 (95% CI 1.015–2.360), *p* = 0.042], and lactate [per 1 mmol/L, hazard ratio 1.049 (95% CI 1.001–1.099), *p* = 0.046] were independently associated with mortality in the multivariable Cox model examining covariates recorded at ICU admission. When cardiac surgery patients were excluded, the significant explanatory variables were sex [female gender hazard ratio 0.321 (95% CI 0.171–0.604), *p* < 0.001], coronary artery disease [hazard ratio 2.917 (95% CI 1.516–5.613), *p* = 0.0014] and APACHE-II [per 1 point, hazard ratio 1.081 (95% CI 1.031–1.135), *p* = 0.0015].

Variables independently associated with mortality in the multivariable Cox model assessing covariates recorded at the initiation of CRRT were lactate [per 1 mmol/L, hazard ratio 1.202 (95% CI 1.137–1.271), *p* < 0.0001], age [per year, hazard ratio 1.032 (95% CI 1.012–1.053), *p* = 0.0014], peripheral artery disease [hazard ratio 2.248 (95% CI 1.260–4.010), *p* = 0.006], creatinine [per 1 mmol/L, hazard ratio 0.997 (95% CI 0.995–1.000), *p* = 0.016], APACHE-II [per 1 point, hazard ratio 1.043 (95% CI 1.006–1.081), *p* = 0.02], and hemoglobin [per 1 mmol/L, hazard ratio 1.015 (95% CI 1.002–1.029), *p* = 0.029]. Fluid balance at CRRT initiation was not associated with mortality [per liter, hazard ratio 1.015 (95% CI 0.979–1.052), *p* = 0.41]. When cardiac surgery patients were excluded from the analysis the model results remained essentially the same and significant explanatory variables were lactate [per 1 mmol/L, hazard ratio 1.202 (95% CI 1.111–1.300), *p* < 0.0001], age [per year, hazard ratio 1.034 (95% CI 1.003–1.066), *p* = 0.003], coronary artery disease [hazard ratio 2.881 (95% CI 1.477–6.619), *p* = 0.0019], creatinine [per 1 mmol/L, hazard ratio 0.996 (95% CI 0.994–0.999), *p* = 0.0075], APACHE-II [per 1 point, hazard ratio 1.071 (95% CI 1.024–1.119), *p* = 0.0025] and hemoglobin [per 1 mmol/L, hazard ratio 1.015 (95% CI 1.002–1.029), *p* = 0.029]. In cardiac surgery patients, duration of cardiopulmonary bypass [per 1 min, hazard ratio 1.003 (95% CI 0.999–1.007), *p* = 0.18] or operation type [compared to CABG only, *p* > 0.10 for all comparisons] were not associated with mortality in the univariate cox models.

**Fig. 3:**
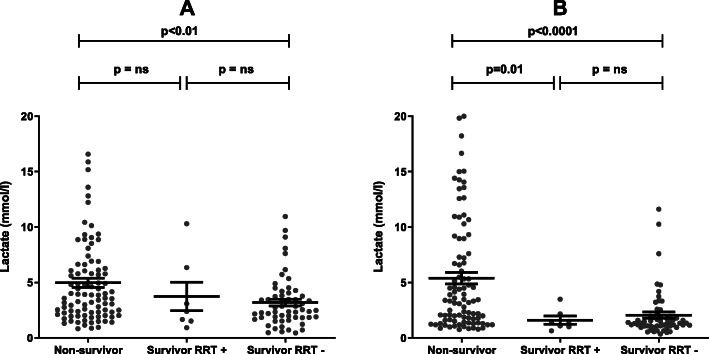
The relationship of lactate at ICU admission (**A**) and at CRRT initiation (**B**) to 90-day survival and renal prognosis

Median (IQR) creatinine was 88 (65–115) μmol/L and eGFR 83 (72–101) mL/min/1.73 m^2^ at 1 year after discharge for the patients who survived. Creatinine was significantly higher and eGFR lower at 90 days (*p* < 0.001 for both) and at 1-year of follow-up (*p* < 0.005 for both) compared to baseline values. Seven and four patients, respectively, required maintenance dialysis at 90 days and at 6 months after ICU discharge. Figure 4 shows the lactate values at ICU admission and at CRRT initiation in non-survivors, survivors on maintenance dialysis, and survivors without maintenance dialysis, respectively, at 90 days following ICU discharge (Fig. 3).

## Discussion

Our current results show that mortality is high in mixed surgical patients with perioperative AKI requiring CRRT. Mortality rates at 90 days were similar across different surgical groups. Blood lactate measured both at ICU admission and at the start of CRRT was independently associated with mortality in the multivariable Cox proportional hazards models. Patients with a lactate level of > 4 mmol/l at CRRT initiation had an ICU mortality of 77%. Lactate at CRRT initiation remained independently associated with mortality when cardiac surgery patients were excluded from the analysis.

Most previous studies on mortality and associated risk factors in postoperative CRRT have concentrated merely on cardiac surgery patients. Recent studies have shown that early-onset hyperlactatemia is associated with an adverse outcome after cardiac surgery (Minton & Sidebotham, 2017; Haas et al., 2016). However, data on the association between hyperlactatemia and overall mortality in a broader spectrum of surgical patients requiring CRRT are scarce. Only a single previous study has reported an association between 90-day mortality and lactate at the start of RRT in a cohort of mixed surgical patients (Lin et al., 2009). Our current results show that postoperative patients who perish during ICU care have higher blood lactate at ICU admission as well as at CRRT initiation compared to survivors and lactate is independently associated with overall mortality. Median lactate at ICU admission was 4.2 mmol/l in non-survivors compared to 2.5 mmol/l in survivors. ICU mortality rate more than doubled in patients with mild lactatemia (2–4 mmol/l) (mortality 50%) and almost quadrupled in patients with lactate exceeding 4 mmol/L (mortality 77%) in comparison to patients with normal lactate at CRRT initiation (mortality 21%), respectively. To our best knowledge, the present study is the first to report lactate thresholds in association with mortality in a broad surgical population requiring CRRT.

Pre-existing comorbidities such as coronary artery disease, diabetes, and peripheral artery disease are common among patients undergoing major surgery and predispose the patient to a greater risk for developing AKI (Biteker et al., 2014). In the current study, ICU non-survivors were older, more often women, and more often had coronary artery disease compared to survivors. Also, the SAPS II, APACHE II, and peak SOFA scores were higher in ICU non-survivors, indicating higher morbidity. However, in the multivariate Cox proportional hazards analyses, only APACHE-II, age, coronary artery disease, and peripheral artery disease remained significantly associated with mortality.

Increase in serum creatinine is the leading biomarker for AKI. According to Kidney Disease Improving Global Outcomes (KDIGO) criteria, AKI is defined as increased serum creatinine (Cr × ≥ 1.5 from the baseline or ≥ 0.3 mg/dl increase within 48 h). Urine output (< 0.5 ml/kg/h) for 6–12 h or and decreased intraoperative urine output (< 0.5 ml/kg/h) have been shown to be associated with postoperative AKI (Myles et al., 2019). In the present study, higher creatinine at the start of CRRT was negatively associated with ICU mortality, which is in line with previous studies (Lin et al., 2009). Although creatinine is useful for classification of AKI, our results together with previous data suggest that higher creatinine at the start of CRRT is not associated with increased mortality risk in AKI patients.

A recent study investigated survival after initiation of CRRT in a cohort of 108 mixed surgical ICU patients and demonstrated that each day of CRRT was associated with 39% higher risk of death in the general surgical patients (Tatum et al., 2017). Another study compared the timing of RRT in postoperative surgical AKI patients. The study demonstrated an in-hospital mortality of 48–67% and a U-shaped association between the time to RRT initiation and mortality (Shiao et al., 2012). The ICU mortality (42.7%) as well as 90-day mortality (58.0%) in the present study was comparable with previous studies (Lin et al., 2009; Tatum et al., 2017; Shiao et al., 2012). We did not observe any difference in the timing of CRRT initiation between survivors and non-survivors. Our findings are in line with a recent large multicenter study (STandard versus Accelerated Initiation of Renal Replacement Therapy in Acute Kidney Injury trial), which found no difference in the risk of death at 90 days between early versus late initiation of CRRT in critically ill AKI patients. (STARRT-AKI group 2020). Notably, the delay to CRRT initiation was very short in the present study.

We observed no difference in the fluid balance at CRRT initiation between survivors and non-survivors. This may be partly related to the varying delay between the operation and CRRT initiation. During the perioperative period, urine output might be reduced in the absence or presence of AKI with or without fluid responsiveness. Therefore, fluids should be administered carefully to avoid hypo- and hypervolemia. The KDIGO guidelines encourage to use preventive strategies in high-risk patients, which include optimization of hemodynamics, restoration of the circulating volume, institution of functional hemodynamic monitoring, and avoidance of nephrotoxic agents and hyperglycemia (Zarbock et al., 2018).

The limitations of this study pertain to its retrospective design and limited sample size. Due to the large number of surgical patients treated in our ICU incidence of perioperative AKI was not evaluated in this study. The follow-up time was extended to 10 years, as only fewer than 2% of surgical patients without prior maintenance dialysis require RRT postoperatively in our center. Since data of the current study were collected at a single center, the results may not apply to other institutions. Unfortunately, operation room data was not systematically recorded electronically at our center during the study period in terms of intraoperative fluid balance (including all fluid losses) and partly concerning blood pressure and therefore, we are unable to report these data reliably and assess their effects on patient mortality in the cohort. However, the ICU patients at our center are documented very meticulously, and the problem of missing values is rather nonexistent for the ICU period. The incidence of CRRT in surgical ICU patients was comparable to previous reports (Mizota et al., 2018; Long et al., 2016). Nevertheless, the findings were quite distinct, and a limited sample size is not likely to detract from the validity of the main findings of this study concerning the risk factor associations with mortality.

## Conclusions

Our study demonstrates that mortality is very high in mixed surgical patients with postoperative AKI requiring CRRT. Lactate at the ICU admission and at the onset of CRRT is independently associated with poor survival in this patient group.

## Supplementary Information

**Additional file 1:.** Supplemental Table 1. Specific antimicrobial regimens used in patients with diagnosed sepsis

## Data Availability

Data that supported the findings of this study are available from the datasets of the Department of Anesthesiology and Intensive Care and the Informatics Department of Turku University Hospital on reasonable request and after permission from the Turku University Clinical Research Center scientific review board and the Hospital district of Southwest Finland.

## Abbreviations

AKI: Acute kidney injury
APACHE-II: Acute Physiology and Chronic Health Evaluation (APACHE-II) II score
CRRT: Continuous renal replacement therapy
eGFR: Estimated glomerular filtration rate
ICU: Intensive care unit
IQR: Inter-quartile range
KDIGO: Kidney Disease Improving Global Outcomes
PaO2/FiO2-ratio: Partial pressure of oxygen in arterial blood/fraction of inspired oxygen-ratio
SAPS: Simplified Acute Physiology Score
SD: Standard deviation
SOFA: Sequential Organ Failure Assessment score
