# Supplementary material for: Mortality and associated risk factors in perioperative acute kidney injury treated with continuous renal replacement therapy
Source: Perioper Med (Lond). 2021 Dec 14;10:57. doi: 10.1186/s13741-021-00227-y (PMC8670067; doi:10.1186/s13741-021-00227-y)
Supplement: Supplementary file 1 — Additional file 1:. Supplemental Table 1. Specific antimicrobial regimens used in patients with diagnosed sepsis [file 13741_2021_227_MOESM1_ESM.docx]

| **Antimicrobial regimen** | **Specific treatment** |
| --- | --- |
| Meropenem | 23 (19%) |
| Piperacillin/Tazobactam | 13 (11%) |
| Fluconazole | 11 (9%) |
| Cloxacillin | 9 (7%) |
| Clindamycin | 8 (7%) |
| Vancomycin | 8 (7%) |
| Imipenem | 7 (6%) |
| Metronidazole | 6 (5%) |
| Rifampicin | 6 (5%) |
| Tigecyclin | 5 (4%) |
| Anidulafungin | 4 (3%) |
| Cefuroxime | 3 (2%) |
| Levofloxacin | 3 (2%) |
| Micafungin | 3 (2%) |
| Moxifloxacin | 3 (2%) |
| Penicillin G | 3 (2%) |
| Ceftriaxone | 2 (2%) |
| Gentamycine | 2 (2%) |
| Daptomycin | 1 (1%) |
| Echinocandin | 1 1%) |
| Linezolide | 1 (1%) |

**Supplemental Table 1.** Spesific antimicrobial regimens used in patients with diagnosed sepsis
